# The Effect of Spontaneous LH Surges on Pregnancy Outcomes in Patients Undergoing Letrozole-HMG IUI: A Retrospective Analysis of 6,285 Cycles

**DOI:** 10.3389/fendo.2022.880538

**Published:** 2022-05-04

**Authors:** Shutian Jiang, Li Chen, Yining Gao, Qianwen Xi, Wenzhi Li, Xinxi Zhao, Yanping Kuang

**Affiliations:** Department of Assisted Reproduction, Shanghai Ninth People’s Hospital, Shanghai Jiaotong University School of Medicine, Shanghai, China

**Keywords:** intrauterine insemination, letrozole, spontaneous LH surge, clinical pregnancy rate, infertility

## Abstract

**Background:**

To date, no consensus has been reached on whether to wait for spontaneous luteinizing hormone (LH) surge to occur or to trigger ovulation regardless of the presence of an LH surge for achieving higher success rate in intrauterine insemination (IUI) cycles. Therefore, we hope to investigate the effect of the presence of a spontaneous LH surge on pregnancy outcomes in letrozole–human menopausal gonadotropin (LE-HMG) IUI cycles.

**Methods:**

In this retrospective cohort study, a total of 6,285 LE-HMG IUI cycles were included between January 2010 and May 2021. Cycles were categorized into three groups: the trigger + LH surge group, the trigger only group, and the LH surge only group. The primary outcome measure was the clinical pregnancy rate. A logistic regression analysis was performed to explore other risk factors affecting the clinical pregnancy rate.

**Results:**

No significant differences were observed in biochemical pregnancy rate (*P* =0.640), clinical pregnancy rate (*P* =0.702), ongoing pregnancy rate (*P* =0.842), and live birth rate (*P* =0.951) among the three groups. The binary logistic regression analysis also confirmed that the existence of an LH surge was not associated with clinical pregnancy. There was a difference in ectopic pregnancy rates (*P* =0.045), but logistic regression showed that the presence of a spontaneous LH surge has no association with ectopic pregnancy. Nonetheless, patients with lead follicles within 18.1-20.0 mm/20.1-22.0 mm and a long duration of LE treatment were less likely to get ectopic pregnant compared with patients with 14.1-16.0 mm lead follicles and shorter LE treatment (OR: 0.142, 95% CI: 0.023–0.891, *P* =0.037; OR: 0.142, 95% CI: 0.022–0.903, *P* =0.039; OR: 0.445, 95% CI: 0.235–0.840, *P* = 0.013).

**Conclusions:**

The presence of a spontaneous LH surge in triggered LE-HMG IUI cycles does not appear to improve pregnancy rates. Thus, we suggest that waiting for an LH surge to occur is not necessary in triggered LE-HMG IUI cycles.

## Background

Intrauterine insemination (IUI) is considered as a first-line treatment for infertility, including a broad range of indications (1, 2). Combining ovarian stimulation (OS) with IUI has been proven to be an effective method. In 2018, a review concluded that it had become clear that IUI–OS is a first-line treatment option for mild male and unexplained infertility (3). Furthermore, a recent systematic review in 2020 claimed and demonstrated that treatment with IUI-OS probably resulted in a higher cumulative live birth rate compared to treatment with IUI in a natural cycle, as well as expectant management without OS (4). Many factors are associated with the success of IUI. Among them, the timing of insemination is known as one of the most important ones.

Timing IUI is usually achieved by monitoring an LH surge or by triggering ovulation. In IUI cycles, human chorionic gonadotropin (hCG) is widely accepted for oocyte maturation and ovulation triggering. Other methods for triggering include the administration of a gonadotrophin-releasing hormone agonist (GnRHa) or a combination of GnRHa and hCG. IUI combined with ovulation triggering is more versatile, requires less LH monitoring, and makes timing for IUI much easier, which has led to this method.

Previous studies have reported conflicting results on which is the best method for IUI timing (3, 5). Furthermore, there is no consensus on whether to wait for a spontaneous LH surge to occur or to trigger ovulation regardless of the presence of an LH surge. A retrospective study by Taerk et al. showed that a higher clinical pregnancy rate was achieved with hCG administration compared with a spontaneous serum LH surge in controlled ovarian hyperstimulation IUI cycles. Moreover, this effect was amplified by receiving hCG in the presence of an LH surge (6). Of note, the difference in clinical pregnancy rates resulted mostly from a subgroup analysis of gonadotropin controlled ovarian stimulation (COH) cycles rather than letrozole (LE) or clomiphene citrate (CC) cycles. Another similar study by Mitwally et al. also reported that hCG treatment was associated with higher pregnancy rates regardless of stimulation protocol, but awaiting for an LH surge only benefited patients undergoing CC-IUI cycles (7). In a randomized clinical trial (RCT) of Kyrou et al., spontaneous LH surge was associated with significantly higher ongoing pregnancy rates compared with the administration of hCG in patients undergoing IUI in natural cycles (8). However, Awonuga and Govindbhai found no significant difference in pregnancy rates between the patients given hCG before and after an endogenous LH surge (9). A systematic review also found no evidence of a difference between the hCG injection group and the LH surge group in the rates of pregnancy (10).

The intent of the current study was to investigate whether the presence of a spontaneous LH surge benefits clinical pregnancy rates in triggered, combined LE-HMG IUI cycles.

## Materials and Methods

### Study Population and Design

This retrospective cohort study was conducted at the Department of Assisted Reproduction of the Ninth People’s Hospital of Shanghai Jiao Tong University School of Medicine between January 2010 and May 2021.

First of all, all the medical histories of the couples were collected through inquiries. Then, all the couples underwent basic infertility tests, including cycle day (CD) 3 follicle-stimulating hormone (FSH), luteinizing hormone (LH), estradiol (E2), progesterone (P), anti-Müllerian hormone (AMH), and transvaginal ultrasound (TVS) of the pelvis. Furthermore, the evaluations of the couples’ physical parameters were performed including heights and weights. Moreover, hysteroscopy for the female partner and semen analyses for the male partner were also completed before the individuals’ therapeutic protocols were determined.

Based on these test results, the inclusion criteria of our study were made as follows: female age <40 years, CD 3 FSH <10 mIU/ml, patent fallopian tubes and a normal uterine cavity according to hysteroscopy, and a male partner with a total sperm concentration of >10 million sperm/ml, a total post-wash sperm count>2 million, and a normal morphology ≥4% (11). In addition, only patients who had one of the following infertility causes were included: ovulatory dysfunction, unexplained factors, mild male factors, and sexual dysfunction. Furthermore, only the first three cycles were recruited if a patient went through more than three cycles of treatment.

Cycles with incomplete medical records were excluded. Patients undergoing ovulation stimulation with drugs other than LE and human menopausal gonadotropin (HMG) were also excluded.

The study protocol was approved by the hospital’s Ethics Committee (Institutional Review Board), and the written informed consent of all couples was required before the treatments (every patient provided their informed consent for our collection and analysis of their treatment-related data with the start of their IUI cycles).

### Ovarian Stimulation Protocols

OSs usually started on CD 3 of menstrual cycles, when TVS were performed and serum FSH, LH, E2, and P were assayed. Patients were given 2.5 or 5 mg LE (Jiangsu Hengrui Medicine Co., Lianyungang, China) orally once daily for 3–5 days. The LE dosage and duration were determined according to the patient’s body mass index (BMI) and length of the menstrual cycle. Generally, the longer the menstrual cycle and the greater the BMI value, the larger the dosage and the longer the duration of LE. From CD 10, routine TVS and hormonal measurements [chemiluminescence (Abbott Biologicals B.V., Olst, the Netherlands)] were performed every 2 days to monitor follicular growth. Once the leading follicle size reached 10 mm in diameter, 75 IU/day HMG (Anhui Fengyuan Pharmaceutical Co., China) was added by intramuscular injection onwards. The duration of HMG varied according to the follicle response. Once a lead follicle reached a mean diameter of 18 mm, trigger was applied. In addition, when a lead follicle reached a mean diameter of 14 mm combined with a slightly elevated LH (10 mIU/ml<LH ≤ 15 mIU/ml), it was also conceived as a criterion for trigger. Generally, 5,000 IU hCG (Lizhu Pharmaceutical Trading Co., Zhuhai, China) or 0.1 mg triptorelin (decapeptyl; Ferring Pharmaceuticals, Saint-Prex, Switzerland) or a combination of the two was administered to induce ovulation, depending on the preference of the doctors in charge. If a markedly elevated LH [LH>15 mIU/ml (12, 13)] occurred, namely, a spontaneous LH surge was detected, we applied trigger drugs immediately or might not have used any medications for trigger, the decision of which was also based on the preference of the doctors in charge. The IUI operation was performed within 24 h after the observed LH surge. In these cases, even though the LH surge was spontaneously generated instead of artificially induced, we called that day the “trigger day.” Notably, if a premature LH surge occurred when the leading follicle size is less than 14 mm in diameter, the cycle would be canceled; meanwhile, cycles with more than 3 dominant follicles were also canceled.

### Sperm Preparation and Insemination

After 2–3 days of abstinence, semen samples were obtained through masturbation and then liquefied for 15–20 min. After that, the sample was washed with 3-layer density gradient centrifugation using Isolate (Irvine Scientific, Santa Ana, California, USA). Insemination was performed on the next day of the confirmation of a spontaneous LH surge (LH>15 mIU/ml), around 24 h after the administration of trigger medicines, or if the detection of a spontaneous LH surge was absent (LH ≤ 15 mIU/ml), the insemination was performed about 38–40 h after the trigger, both with a soft catheter (Cook Group, Bloomington, Indiana, USA). Only one insemination was performed per cycle.

### Outcome Variables and Definitions

The primary outcome measure of the study was the clinical pregnancy rate (CPR). The secondary measures included the biochemical pregnancy rate, ongoing pregnancy rate, live birth rate, miscarriage rate, ectopic pregnancy rate, and multiple pregnancy rate.

The clinical pregnancy was confirmed by the ultrasonic demonstration of at least one intrauterine gestational sac with fetal cardiac activity 6 weeks after IUI. The biochemical pregnancy was defined by a serum βhCG concentration > 5 mIU/ml collected 18 days after insemination. The ongoing pregnancy was referred to a pregnancy of more than 12 weeks of gestational age. The miscarriage was defined as the spontaneous termination of a fetus before 28 weeks of gestation. The live birth was conceived as the delivery of a live infant after 28 weeks of gestation. The ectopic pregnancy denoted the implantation of an embryo outside the uterine cavity. The multiple pregnancy was identified as a pregnancy with more than one fetus.

### Statistical Analysis

All of the cycles were divided into three groups: the trigger + LH surge group (trigger day LH >15 mIU/ml + trigger drugs), the trigger-only group (trigger day LH ≤15 mIU/ml + trigger drugs), and the LH surge-only group (trigger day LH >15 mIU/ml, without the use of trigger drugs). Statistical analysis was performed using the Statistical Package for the Social Sciences (version 26.0; SPSS Inc., Armonk, New York, USA). Continuous variables were presented as mean ± SD. The normality of continuous variables was determined using the Shapiro–Wilk test and Q-Q plots. The variables were compared by one-way analysis of variance (ANOVA) with *post-hoc* analysis or Welch’s t-test as required. Categorical variables were put forward in the number of cases (n) with percentages (%). The comparisons of rates between groups were completed by the chi-square test or Fisher’s exact test as required. Binary logistic regression analysis was used to evaluate the impact of the other factors on the clinical pregnancy rate. P<0.05 was considered statistically significant.

## Results

A total of 5,098 patients who underwent 6,285 IUI cycles from January 2010 to May 2021 were enrolled in this study, as shown in **Supplemental Figure 1**. Among them, 2,588 (41.18%) cycles were categorized into the trigger + LH surge group, 3,487 (55.48%) cycles were categorized into the trigger-only group, and the remaining cycles were classified as the LH surge-only group.

The demographic and basic characteristics of the patients are presented in **Table 1**. No significant differences were observed regarding the female age, male age, male BMI, duration of infertility, infertility type, infertility cause basal hormone level, or rank of IUI attempts among the three groups. Women in the trigger + LH surge group showed the highest BMI value, while those in the LH surge-only group showed the lowest BMI value (22.50 ± 3.56 kg/m^2^ vs. 21.95 ± 5.43 kg/m^2^ vs. 21.41 ± 2.71 kg/m^2^, P<0.001). Another difference obtained from the comparison was that the patients in the trigger + LH surge group displayed higher antral follicle count (AFC), compared with the other two groups (14.25 ± 6.79 vs. 13.45 ± 6.39 vs. 13.09 ± 5.44, P<0.001). For the steroid hormone profiles, the trigger + LH surge group revealed significantly higher basal LH (4.19 ± 2.62 mIU/ml vs. 3.86 ± 1.92 mIU/ml vs. 3.90 ± 1.89 mIU/ml, P<0.001) and lower basal P (0.26 ± 0.12 ng/ml vs. 0.28 ± 0.14 ng/ml vs. 0.26 ± 0.19 ng/ml, P=0.02). The basal E2 and AMH were both comparable.

**Table 1:** The demographic and basic characteristics of patients.

	Group A^c^ Trigger + LH surge (n=2,128)	Group B^c^ Trigger only (n=2,782)	Group C^c^ LH surge only (n=188)	P-value
Female age (years)	31.18 ± 3.61	31.01 ± 3.62	30.87 ± 3.07	0.084
Male age (years)	32.89 ± 4.50	32.85 ± 4.45	32.57 ± 3.92	0.097
Female BMI (kg/m^2^)	22.50 ± 3.56	21.95 ± 5.43	21.41 ± 2.71	<0.001^a^
Male BMI (kg/m^2^)	24.56 ± 3.48	24.55 ± 3.43	24.30 ± 3.34	0.804
Duration of infertility (years)	3.13 ± 2.11	3.17 ± 2.09	3.07 ± 1.72	0.660
Infertility type, n (%)				0.400
Primary	1,439 (67.6)	1,901 (68.3)	136 (72.3)	
Secondary	689 (32.4)	881 (31.7)	52 (27.7)	
Infertility causes, n (%)				0.968
Ovulatory dysfunction	421 (19.8)	539 (19.4)	34 (18.1)	
Sexual dysfunction	116 (5.5)	135 (4.9)	10 (5.3)	
Mild male factor	310 (14.6)	411 (14.8)	28 (14.9)	
Unexplained factor	1281 (60.2)	1697 (61.0)	116 (61.7)	
Antral follicles	14.25 ± 6.79	13.45 ± 6.39	13.09 ± 5.44	<0.001^a^
Basal hormonal level				
FSH (mIU/ml)	5.68 ± 1.31	5.59 ± 1.30	5.65 ± 1.26	0.065
LH (mIU/ml)	4.19 ± 2.62	3.86 ± 1.92	3.90 ± 1.89	<0.001^a^
E_2_ (pg/ml)	34.63 ± 15.01	34.16 ± 15.19	35.71 ± 19.36	0.427
P (ng/ml)	0.26 ± 0.12	0.28 ± 0.14	0.26 ± 0.19	<0.001^b^
AMH (ng/ml)	4.80 ± 3.87	4.51 ± 3.80	4.86 ± 3.88	0.635
Rank of IUI attempts, n (%)				0.094
1st cycle	1,084 (50.9)	1,417 (50.9)	111 (59.0)	
2nd cycle	829 (39.0)	1,111 (39.9)	57 (30.3)	
3rd cycle	215 (10.1)	254 (9.1)	20 (10.6)	

BMI, body mass index; FSH, follicle-stimulating hormone; LH, luteinizing hormone; E_2_, estradiol; P, progesterone; AMH, anti-Müllerian hormone; IUI, intrauterine insemination.

Data are presented as mean ± SD or number of cases (n) with rate (%).

a The value in group A was significantly higher than the values in groups B and C.

b The value in group A was significantly lower than the values in group B.

c IUI was performed around 24 h after the observation of a spontaneous LH surge (LH>15 mIU/ml) both in Group A and Group C, or if the detection of a spontaneous LH surge was absent (LH ≤ 15 mIU/ml), IUI was performed approximately 38–40 h after the trigger in group B.

**Table 2** describes the cycle characteristics and hormone profiles of the three groups of IUI treatment. Among the three groups, the trigger-only group showed the notably longest LE treatment length, highest total LE dose, and shortest cycle duration (*P <*0.001). Meanwhile, the patients in the trigger-only group obviously had the thinnest endometrium (*P <*0.001). There was a general trend that the patients in the trigger-only group presented the most lead follicles, while their lead follicle size was generally the largest (*P <*0.001). However, in terms of the sexual hormone levels on the trigger day, the E2 concentrations and the average E2 value (the ratio of E2 to the dominant follicle count) were distinctly lowest in the trigger-only group (*P <*0.001). Naturally, the concentrations of LH and P were both higher for the patients with a spontaneous LH surge (in the trigger + LH surge group and LH surge-only group), given the appearance of premature LH surge in this group (*P <*0.001). In addition, differences could be detected in the distribution of trigger regimen between the two groups with the use of trigger drugs, namely, a gonadotrophin-releasing hormone agonist (GnRHa) was more often prescribed to patients without a spontaneous LH surge, while hCG was usually administrated to those with a spontaneous LH surge (*P <*0.001).

**Table 2:** The cycle characteristics and hormone profiles of patients undergoing IUI.

	Group A^f^ Trigger + LH surge (n=2588)	Group B^f^ Trigger only (n=3487)	Group C^f^ LH surge only (n=210)	P-value
Total LE dose per cycle (mg)	14.90 ± 6.34	15.91 ± 5.65	15.67 ± 5.36	<0.001^a^
Length of LE treatment (days)	4.45 ± 1.14	5.01 ± 1.13	4.72 ± 1.13	<0.001^b^
Cycle duration (days)	11.75 ± 2.80	10.45 ± 2.40	10.81 ± 3.00	<0.001^c^
Endometrial thickness at triggering (mm)	10.15 ± 2.26	9.74 ± 2.21	9.85 ± 2.18	<0.001^c^
Hormone level on trigger day				
E_2_ (pg/ml)	292.45 ± 209.39	248.70 ± 153.41	273.41 ± 191.66	<0.001^d^
Average E_2_ (pg/ml)	219.04 ± 154.76	168.15 ± 93.68	186.54 ± 106.31	<0.001^e^
LH (mIU/ml)	21.73 ± 13.27	5.40 ± 2.41	18.12 ± 8.78	<0.001^e^
P (ng/ml)	0.64 ± 0.44	0.29 ± 0.13	0.46 ± 0.40	<0.001^e^
Number of lead follicles				<0.001
1	1,685 (65.1)	1,834 (52.6)	120 (57.1)	
2	699 (27.0)	1169 (33.5)	67 (31.9)	
3	204 (7.9)	484 (13.9)	23 (11.0)	
Lead follicle size, n (%)				<0.001
14.1-16.0 mm	89 (3.4)	11 (0.3)	4 (1.9)	
16.1-18.0 mm	381 (14.7)	130 (3.7)	37 (17.6)	
18.1-20.0 mm	957 (37.0)	1467 (42.1)	102 (48.6)	
20.1-22.0 mm	1,161 (44.9)	1,879 (53.9)	67 (31.9)	
Trigger for ovulation, n (%)				<0.001
5,000 IU hCG	877 (33.9)	691 (19.8)	/	
0.1 mg GnRHa	1,502 (58.0)	2,532 (72.6)	/	
5,000 IU hCG +0.1 mg GnRHa	209 (8.1)	264 (7.6)	/	

LE, letrozole; E_2_, estradiol; average E_2_, the ratio of E_2_ to dominant follicle count; LH, luteinizing hormone; P, progesterone.

Data are presented as mean ± SD or number of cases (n) with rate (%).

a The value in group A was significantly lower than the value in group B.

b The value in group A was significantly lower than the values in groups B and C. The value in group C was significantly lower than the value in group B.

c The value in group A was significantly higher than the values in groups B and C.

d The value in group A was significantly higher than the value in group B.

e The value in group A was significantly higher than the values in groups B and C. The value in group C was significantly higher than the value in group B.

f IUI was performed around 24 h after the observation of a spontaneous LH surge (LH>15 mIU/ml) both in group A and group C, or if the detection of a spontaneous LH surge was absent (LH ≤ 15 mIU/ml), IUI was performed approximately 38–40 h after the trigger in group B.

The pregnancy outcomes of the IUI cycles are detailed in **Table 3**. The pregnancy outcomes were generally similar among the three groups in terms of the biochemical pregnancy rate, clinical pregnancy rate, ongoing pregnancy rate, miscarriage rate, and live birth rate. Meanwhile, the multiple pregnancy rate also seemed to be comparable among the three groups. Nonetheless, it was worth mentioning that the ectopic pregnancy rate in the trigger + LH surge group was significantly higher than that of the trigger-only group (2.8% vs. 0.7% vs. 0%, P=0.045). There was no case of ectopic pregnancy in the LH surge-only group, but considering the small sample size in this group, together with the low incidence of ectopic pregnancy, this result might be a result of chance.

**Table 3:** The pregnancy outcomes of patients undergoing IUI.

	Group A^a^ Trigger + LH surge (n=2,588)	Group B^a^ Trigger only (n=3,487)	Group C^a^ LH surge only (n=210)	P -value
Clinical pregnancy	424 (16.4)	545 (15.6)	35 (16.7)	0.702
Biochemical pregnancy	460 (17.8)	601 (17.2)	41 (19.5)	0.640
Ongoing pregnancy	371 (14.3)	488 (14.0)	32 (15.2)	0.842
Live birth	356 (13.8)	474 (13.6)	30 (14.3)	0.951
Ectopic pregnancy	12/424 (2.8)	4/545 (0.7)	0	0.045
Multiple pregnancy	31/424 (7.3)	49/545 (9.0)	3/35 (8.6)	0.659
Triplet	1/424 (0.2)	0	0	0.473
Miscarriage	68/424 (16.0)	71/545 (13.0)	5/35 (14.3)	0.431

Data are presented as cases (n) with rate (%).

a IUI was performed approximately 24 h after the observation of a spontaneous LH surge (LH>15 mIU/ml) both in group A and group C, or if the detection of a spontaneous LH surge was absent (LH ≤ 15 mIU/ml), IUI was performed approximately 38–40 h after the trigger in group B.

**Table 4** exhibits the results of multiple binary logistic regression analyses accounting for the risk factors that may have an impact on clinical pregnancy outcomes. The results revealed an obviously significant increase in the probability of clinical pregnancy with an increase in the cycle duration (OR: 1.043, 95% CI: 1.006-1.082, *P* = 0.023). At the same time, the endometrial thickness at triggering was proven to be positively associated with the clinical pregnancy rate (OR: 1.064, 95% CI: 1.024-1.105, *P* = 0.002), while the duration of infertility was demonstrated to be associated with a decreased probability of clinical pregnancy (OR 0.936, 95% CI 0.895-0.978, *P* = 0.003). Compared with patients with only one lead follicle at the time of triggering, patients with two or three lead follicles were 2.000 or 2.075 times more likely to get clinically pregnant (OR: 2.000, 95% CI: 1.522-2.628, *P <*0.001; OR: 2.075, 95% CI: 1.324-3.246, *P* =0.001). Moreover, patients with sexual dysfunction had 2.082 times more potential to achieve clinical pregnancy than patients with ovulatory dysfunction (OR: 2.082, 95% CI: 1.447-2.996, *P <*0.001). Additionally, compared with patients undergoing their first IUI cycles, patients attempting a third IUI cycle were associated with a decrease in the likelihood of clinical pregnancy (OR: 0.830, 95% CI: 0.691-0.997, *P* = 0.047). In particular, it was found that whether the LH surge was spontaneous or not did not affect the clinical pregnancy rate.

**Table 4:** Binary logistic regression to account for variables associated with clinical pregnancies after undergoing LE ovulation induction with IUI (n=6,285).

Clinical Pregnancy	Adjusted OR (95% CI)	P-value
Total LE dose per cycle (mg)	1.017 (0.999-1.036)	0.069
Length of LE treatment (days)	0.945 (0.863-1.034)	0.219
Cycle duration (days)	1.043 (1.006-1.082)	0.023*
Trigger day E2 level (pg/ml)	0.999 (0.998-1.000)	0.281
Trigger day average E2 level (pg/ml)	1.001 (1.000-1.003)	0.053
Trigger day LH level (mIU/ml)	1.004 (0.994-1.013)	0.435
Trigger day P level (ng/ml)	0.902 (0.677-1.201)	0.481
Endometrial thickness at triggering (mm)	1.064 (1.024-1.105)	0.002*
No. of lead follicles (1 vs. 2)	2.000 (1.522-2.628)	<0.001*
No. of lead follicles (1 vs. 3)	2.075 (1.324-3.246)	0.001*
Female age (years)	0.993 (0.958-1.029)	0.709
Male age (years)	1.005 (0.977-1.034)	0.725
Female BMI (kg/m^2^)	1.027 (1.001-1.053)	0.043*
Male BMI (kg/m^2^)	1.008 (0.984-1.032)	0.538
Infertility type (primary vs. secondary)	1.133 (0.942-1.363)	0.185
Infertility cause (ovulatory dysfunction vs. sexual dysfunction)	2.082 (1.447-2.996)	<0.001*
Infertility cause (ovulatory dysfunction vs. male factor)	0.966 (0.711-1.313)	0.827
Infertility cause (ovulatory dysfunction vs. unexplained factor)	0.786 (0.618-0.999)	0.049*
Antral follicles	0.996 (0.983-1.009)	0.557
Rank of IUI attempts (1st vs. 3rd)	0.830 (0.691-0.997)	0.047*
Rank of IUI attempts (2nd vs. 3rd)	1.149 (0.854-1.547)	0.359
Duration of infertility (years)	0.936 (0.895-0.978)	0.003*
5,000 IU hCG vs. 0.1 mg GnRHa	1.029 (0.847-1.249)	0.774
5,000 IU hCG vs. 5,000 IU hCG +0.1mg GnRHa	1.145 (0.834-1.573)	0.403
Follicle size (14.1-16.0 vs. 16.1-18.0 mm)	1.001 (0.545-1.837)	0.998
Follicle size (14.1-16.0 vs. 18.1-20.0 mm)	0.874 (0.492-1.554)	0.648
Follicle size (14.1-16.0 vs. 20.1-22.0 mm)	0.809 (0.455-1.439)	0.471
Trigger + LH surge vs. trigger only	1.113 (0.883-1.402)	0.365
Trigger + LH surge vs. LH surge only	0.817 (0.323-2.064)	0.669

CI, confidence interval; OR, odds ratio.

*P < 0.05.

To further assess the impact of risk factors on the ectopic pregnancy in IUI cycles, a binary logistic analysis was performed, as shown in **Table 5**. The ectopic pregnancy was less likely to occur when the LE treatment length extended (OR: 0.445, 95% CI: 0.235–0.840, *P* = 0.013). As for the lead follicle size on the trigger day, patients with lead follicles within the 18.1-20.0 mm/20.1-22.0 mm interval were less likely to get ectopically pregnant compared with patients with 14.1-16.0 mm lead follicles (OR: 0.142, 95% CI: 0.023–0.891, *P* =0.037; OR: 0.142, 95% CI: 0.022–0.903, *P* =0.039). Notably, it was also demonstrated that whether there was a spontaneous LH surge or not was not associated with the ectopic pregnancy.

**Table 5:** Binary logistic regression to account for variables associated with ectopic pregnancies after undergoing LE ovulation induction with IUI (n =6,285).

Ectopic Pregnancy	Unadjusted OR (95% CI)	P-value
Total LE dose per cycle (mg)	1.120 (0.995-1.261)	0.060
Length of LE treatment (days)	0.445 (0.235-0.840)	0.013*
Cycle duration (days)	0.900 (0.702-1.154)	0.406
Trigger day E2 level (pg/ml)	0.987 (0.969-1.006)	0.174
Trigger day average E2 level (pg/ml)	1.012 (0.991-1.033)	0.273
Trigger day LH level (mIU/ml)	1.006 (0.962-1.052)	0.800
Trigger day P level (ng/ml)	0.713 (0.137-3.712)	0.688
Endometrial thickness at triggering (mm)	1.013 (0.800-1.282)	0.916
No. of lead follicles (1 vs. 2)	5.203 (0.450-60.223)	0.187
No. of lead follicles (1 vs. 3)	14.917 (0.370-602.077)	0.152
Female age (years)	1.106 (0.890-1.375)	0.363
Male age (years)	0.995 (0.840-1.179)	0.957
Female BMI (kg/m^2^)	1.003 (0.884-1.139)	0.958
Male BMI (kg/m^2^)	0.992 (0.849-1.159)	0.915
Infertility type (primary vs. secondary)	1.573 (0.534-4.631)	0.411
Infertility cause (ovulatory dysfunction vs. sexual dysfunction)	0.705 (0.062-8.069)	0.779
Infertility cause (ovulatory dysfunction vs. male factor)	0.397 (0.039-4.060)	0.436
Infertility cause (ovulatory dysfunction vs. unexplained factor)	0.699 (0.174-2.805)	0.614
Antral follicles	1.036 (0.966-1.110)	0.323
Rank of IUI attempts (1st vs. 2nd)	1.405 (0.489-4.039)	0.528
Rank of IUI attempts (1st vs. 3rd)	0.000 (0.000-.)	0.994
Duration of infertility (years)	0.793 (0.575-1.094)	0.157
5,000 IU hCG vs. 0.1 mg GnRHa	0.693 (0.237-2.024)	0.502
5,000 IU hCG vs. 5,000 IU hCG +0.1 mg GnRHa	0.000 (0.000-.)	0.993
Follicle size (14.1-16.0 vs. 16.1-18.0 mm)	0.114 (0.009-1.402)	0.090
Follicle size (14.1-16.0 vs. 18.1-20.0 mm)	0.142 (0.023-0.891)	0.037*
Follicle size (14.1-16.0 vs. 20.1-22.0 mm)	0.142 (0.022-0.903)	0.039*
Trigger + LH surge vs. trigger only	0.428 (0.098-1.869)	0.259
Trigger + LH surge vs. LH surge only	1.178 (0.000-.)	1.000

CI, confidence interval; OR, odds ratio.

*P < 0.05.

## Discussion

This study suggested that the presence of a spontaneous LH surge had no beneficial effect on the pregnancy rates in triggered LE-HMG IUI cycles. In addition, we were among the first to evaluate the effect of a spontaneous LH surge/follicle size on the ectopic pregnancy rate. Our data suggested that there was no evidence of an association between a spontaneous LH surge and ectopic pregnancy. However, patients with smaller follicles (14.1-16.0 mm) might be more likely to get ectopic pregnancy compared with patients with lead follicles within the 18.1-20.0 mm/20.1-22.0 mm interval. We also found that the risk of ectopic pregnancy might be higher with a shorter duration of LE treatment.

Among all the factors contributing to the success of IUI, it has been suggested that the timing of insemination is probably one of the most important ones (3). Our study drew similar conclusions as the previous report by Awonuga and Govindbhai. They did not find any significant difference in the pregnancy rates between the cycles in which hCG was given before or after an LH surge (9). They suggested that waiting for the LH surge to occur in the presence of a mature follicle provided no benefit to patients in IUI cycles. A study by Pittrof et al. also confirmed our results. They reported that presence of a spontaneous LH surge before the administration of hCG was not associated with a successful outcome of IUI (14). However, several studies suggested differently. Taerk et al. and Mitwally et al. both reported that a significantly higher pregnancy rate was achieved if a spontaneous LH surge occurred before hCG administration (6, 7). Nonetheless, these results did not apply to patients receiving LE. Both studies found this difference statistically insignificant with LE-IUI cycles, which was in accordance with our results (6, 7). Also, based on our study, there did not seem to be an association between an hCG/GnRHa treatment and a higher clinical pregnancy rate in LE-HMG IUI cycles, with the rate of 16.4% (424/2,588) in the trigger+ LH surge group, the rate of 15.6% (545/3,487) in the trigger-only group, and the rate of 16.7% (35/210) in the LH surge- only group. Taerk et al. also found no difference in the clinical pregnancy rates among hCG the trigger-only group, LH surge-only group, and LH surge+hCG trigger group in patients receiving LE. However, in their study, only one pregnancy resulted from 11 cycles using LE.

A possible explanation for why waiting for spontaneous LH surge did not apply to LE-HMG IUI cycles is a lower estrogen level. As an aromatase inhibitor, LE induces a reduction of circulating estrogen concentration, which enables the pituitary gland to escape from estrogen feedback (15). Furthermore, a study by Bedaiwy et al. showed that LH was significantly lower on the day of hCG administration in the LE group than the natural group (16). Thus, the detection of an LH surge might not be as accurate in the LE-HMG IUI cycle, especially using urinary LH monitoring. We use serum LH monitoring to avoid such issue as much as possible.

It was suggested in our study that a spontaneous LH surge might not be associated with the ectopic pregnancy, while lead follicles within 14.1-16.0 mm were probably related to a higher risk of ectopic pregnancy. So far, limited literature focusing on these two topics is available. Therefore, we are among the first to report the relationships between a spontaneous LH surge/follicle size and the ectopic pregnancy, the underlying mechanism of which awaits further exploration. Another intriguing finding was that it seemed that the shorter the LE treatment, the higher the risk of ectopic pregnancy. As is known, LE inhibits the synthesis of estrogen, resulting in a significantly lower E2 level. According to some studies, ectopic pregnancy was significantly higher in stimulated cycles or two-follicle HMG cycles, compared with natural cycles for IUI cycles (17, 18). Meanwhile another study showed that the rate of ectopic pregnancy increased with the peak E2 level for in-vitro fertilization (IVF)/intracytoplasmic sperm injection (ICSI) cycles (19). All of these results Indicate that the tubal-uterine environment after OS may be different from the physiological status in the context of a high E2 level, which contributes to abnormal implantation eventually.

In the present study, the existence of an LH surge had also been demonstrated to not be associated with the clinical pregnancy. Meanwhile, the following factors were proven to have an influence on the clinical pregnancy. First, the duration of infertility was found negatively correlated with the clinical pregnancy rate. Hansen et al. and Mahnaz et al. reached a similar conclusion as ours, in which it was found that the duration of infertility ≤5 years was a favorable factor for treatment success in IUI (20, 21). Next, patients undergoing their third IUI cycle/with only one lead follicle were less likely to get clinically pregnant, compared with patients undergoing their second IUI cycles/with two or three lead follicles. These results were in agreement with the previous ones (2, 22–24). Finally, the endometrial thickness was verified to be positively correlated with the clinical pregnancy rate, the conclusion of which was controversial based on previous studies. While Marhar et al. and Liu et al. found that endometrial thickness contributed to the success rate in IUI cycles (25, 26), other studies found no evidence for an association between endometrial thickness and pregnancy rates during IUI (27).

The current study had several strengths. To date, it is the largest cohort study focusing on the occurrence of a spontaneous LH surge in IUI cycles with a sample size of 6,075. Also, serum LH monitoring was used to determine an LH surge, making this process much more accurate than urinary LH monitoring in other studies. In addition, we are among the first to investigate the relationship between a spontaneous LH surge/follicle size and ectopic pregnancy, which provides clinical guidance. However, there were also several limitations that must be noticed. Firstly, this is a retrospective single-center study, but we did our best to execute strict inclusion criteria. It is also worth mentioning that maybe a retrospective design is more suitable for studying spontaneous LH surges compared to RCTs. The reason behind this is that it is impossible to predict when an LH surge will occur and whether there is going to be an LH surge at all. Secondly, the size of the lead follicles might be affected due to the prolonged waiting for an LH surge, which might be a confounding factor. Thirdly, there was heterogeneity in trigger medication and timing in our study. Particularly, patients with spontaneous LH surge were more often triggered with hCG, and those without were more often triggered with a GnRHa, which might have an impact on our results. Fourthly, apart from patients diagnosed as having unexplained infertility and anovulation, we also include mild male factor and sexual dysfunction patients in our study. These might introduce confounding factors into the study, possibly affecting the results. In addition, since we had two outcome measures evaluated in the binary regression analysis (clinical pregnancy and ectopic pregnancy), substantial amounts of multiple comparison bias were probably introduced into the results. Further, well-designed large RCTs could be conducted to confirm our results.

## Conclusion

The presence of a spontaneous LH surge in triggered LE-HMG IUI cycles does not appear to improve pregnancy rates. Moreover, patients with lead follicles between 14.1 and 16.0 mm or a shorter duration of LE treatment may be at a greater risk for ectopic pregnancy. However, a spontaneous LH surge is not associated with ectopic pregnancy. Thus, we suggest that waiting for an LH surge to occur is not necessary in triggered LE-HMG IUI cycles. However, this is a retrospective study, so further well-designed prospective cohort studies will be needed to confirm the conclusion.

## Data Availability Statement

The raw data supporting the conclusions of this article will be made available by the authors, without undue reservation.

## Ethics Statement

The studies involving human participants were reviewed and approved by the Ethics Committee of Shanghai 9th People’s Hospital (Institutional Review Board). Written informed consent for participation was not required for this study in accordance with the national legislation and the institutional requirements.

## Author Contributions

YK contributed to the conception and design of the study. LC and SJ analyzed the data. LC, SJ, and YG drafted the manuscript. QX, WL, and XZ participated critical discussion and revised the manuscript. All authors have reviewed the manuscript and approved the final version.

## Funding

This research was supported by the National Key Research and Development Program of China (2018YFC1003000) and the National Natural Science Foundation of China (81901478).

## Conflict of Interest

The authors declare that the research was conducted in the absence of any commercial or financial relationships that could be construed as a potential conflict of interest.

## Publisher’s Note

All claims expressed in this article are solely those of the authors and do not necessarily represent those of their affiliated organizations, or those of the publisher, the editors and the reviewers. Any product that may be evaluated in this article, or claim that may be made by its manufacturer, is not guaranteed or endorsed by the publisher.

## References

[1] Carson SA Kallen AN. Diagnosis and Management of Infertility: A Review. JAMA (2021) 326(1):65. doi: 10.1001/jama.2021.4788 34228062 PMC9302705

[2] Merviel P Heraud MH Grenier N Lourdel E Sanguinet P Copin H. Predictive Factors for Pregnancy After Intrauterine Insemination (IUI): An Analysis of 1038 Cycles and a Review of the Literature. Fertility Sterility (2010) 93(1):79–88. doi: 10.1016/j.fertnstert.2008.09.058 18996517

[3] Cohlen B Bijkerk A van der Poel S Ombelet W. IUI: Review and Systematic Assessment of the Evidence That Supports Global Recommendations. Hum Reprod Update (2018) 24(3):300–19. doi: 10.1093/humupd/dmx041 29452361

[4] Ayeleke RO Asseler JD Cohlen BJ Veltman-Verhulst SM. Intra-Uterine Insemination for Unexplained Subfertility. Cochrane Database Syst Rev (2020) 3(3):Cd001838. doi: 10.1002/14651858.CD001838.pub6 32124980 PMC7059962

[5] Kosmas IP Tatsioni A Fatemi HM Kolibianakis EM Tournaye H Devroey P. Human Chorionic Gonadotropin Administration vs. Luteinizing Monitoring for Intrauterine Insemination Timing, After Administration of Clomiphene Citrate: A Meta-Analysis. Fertil Steril (2007) 87(3):607–12. doi: 10.1016/j.fertnstert.2006.10.003 17173907

[6] Taerk E Hughes E Greenberg C Neal M Amin S Faghih M. Controlled Ovarian Hyperstimulation With Intrauterine Insemination Is More Successful After r-hCG Administration Than Spontaneous LH Surge. J Reprod Infertil (2017) 18(3):316–22. PMC5641441 29062796

[7] Mitwally MF Abdel-Razeq S Casper RF. Human Chorionic Gonadotropin Administration is Associated With High Pregnancy Rates During Ovarian Stimulation and Timed Intercourse or Intrauterine Insemination. Reprod Biol Endocrinol (2004) 2:55. doi: 10.1186/1477-7827-2-55 15239837 PMC479701

[8] Kyrou D Kolibianakis EM Fatemi HM Grimbizis GF Theodoridis TD Camus M. Spontaneous Triggering of Ovulation Versus HCG Administration in Patients Undergoing IUI: A Prospective Randomized Study. Reprod BioMed Online (2012) 25(3):278–83. doi: 10.1016/j.rbmo.2012.05.005 22796236

[9] Awonuga A Govindbhai J. Is Waiting for an Endogenous Luteinizing Hormone Surge and/or Administration of Human Chorionic Gonadotrophin of Benefit in Intrauterine Insemination? Hum Reprod (1999) 14(7):1765–70. doi: 10.1093/humrep/14.7.1765 10402385

[10] Cantineau AE Janssen MJ Cohlen BJ Allersma T. Synchronised Approach for Intrauterine Insemination in Subfertile Couples. Cochrane Database Syst Rev (2014) (12), CD006942. doi: 10.1002/14651858.CD006942.pub3 25528596 PMC11182568

[11] Starosta A Gordon CE Hornstein MD. Predictive Factors for Intrauterine Insemination Outcomes: A Review. Fertil Res Pract (2020) 6(1):23. doi: 10.1186/s40738-020-00092-1 33308319 PMC7731622

[12] Meyer L Murphy LA Gumer A Reichman DE Rosenwaks Z Cholst IN. Risk Factors for a Suboptimal Response to Gonadotropin-Releasing Hormone Agonist Trigger During *In Vitro* Fertilization Cycles. Fertil Steril (2015) 104(3):637–42. doi: 10.1016/j.fertnstert.2015.06.011 26149355

[13] Guo H Li J Shen X Cong Y Wang Y Wu L. Efficacy of Different Progestins in Women With Advanced Endometriosis Undergoing Controlled Ovarian Hyperstimulation for *In Vitro* Fertilization-A Single-Center Non-Inferiority Randomized Controlled Trial. Front Endocrinol (Lausanne) (2020) 11:129. doi: 10.3389/fendo.2020.00129 32265834 PMC7103634

[14] Pittrof RU Shaker A Dean N Bekir JS Campbell S Tan S-L. Success of Intrauterine Insemination Using Cryopreserved Donor Sperm Is Related to the Age of the Woman and the Number of Preovulatory Follicles. J Assisted Reprod Genet (1996) 13(4):310–4. doi: 10.1007/BF02070144 8777345

[15] Garcia-Velasco JA. The Use of Aromatase Inhibitors in *In Vitro* Fertilization. Fertility Sterility (2012) 98(6):1356–8. doi: 10.1016/j.fertnstert.2012.09.042 23062732

[16] Bedaiwy MA Abdelaleem MA Hussein M Mousa N Brunengraber LN Casper RF. Hormonal, Follicular and Endometrial Dynamics in Letrozole-Treated Versus Natural Cycles in Patients Undergoing Controlled Ovarian Stimulation. Reprod Biol Endocrinol (2011) 9(1):83. doi: 10.1186/1477-7827-9-83 21693028 PMC3131247

[17] Bu Z Xiong Y Wang K Sun Y. Risk Factors for Ectopic Pregnancy in Assisted Reproductive Technology: A 6-Year, Single-Center Study. Fertility Sterility (2016) 106(1):90–4. doi: 10.1016/j.fertnstert.2016.02.035 27001382

[18] Li S He Y Cao M Liu H Liu J. Low-Dose Human Menopausal Gonadotrophin Versus Natural Cycles in Intrauterine Insemination for Subfertile Couples With Regular Menstruation. J Ovarian Res (2020) 13(1):36. doi: 10.1186/s13048-020-00638-3 32247312 PMC7129328

[19] Wang J Wei Y Diao F Cui Y Mao Y Wang W. The Association Between Polycystic Ovary Syndrome and Ectopic Pregnancy After *In Vitro* Fertilization and Embryo Transfer. Am J Obstetrics Gynecology (2013) 209(2):139.e1–.e9. doi: 10.1016/j.ajog.2013.05.007 23659986

[20] Ashrafi M Daghighi S Pourasghari P Zolfaghari Z. The Role of Infertility Etiology in Success Rate of Intrauterine Insemination Cycles: An Evaluation of Predictive Factors for Pregnancy Rate. Int J Fertil Steril (2013) 7(2):100–7. PMC3850345 24520471

[21] Hansen KR He ALW Styer AK Wild RA Butts S Engmann L. Predictors of Pregnancy and Live-Birth in Couples With Unexplained Infertility After Ovarian Stimulation–Intrauterine Insemination. Fertility Sterility (2016) 105(6):1575–83.e2. doi: 10.1016/j.fertnstert.2016.02.020 26949110 PMC4893990

[22] Plosker SM Jacobson W Amato P. Predicting and Optimizing Success in an Intra-Uterine Insemination Programme. Hum Reprod (1994) 9(11):2014–21. doi: 10.1093/oxfordjournals.humrep.a138385 7868666

[23] Stone BA Vargyas JM Ringler GE Stein AL Marrs RP. Determinants of the Outcome of Intrauterine Insemination: Analysis of Outcomes of 9963 Consecutive Cycles. Am J Obstetrics Gynecology (1999) 180(6):1522–34. doi: 10.1016/S0002-9378(99)70048-7 10368500

[24] Custers IM Steures P Hompes P Flierman P van Kasteren Y van Dop PA. Intrauterine Insemination: How Many Cycles Should We Perform? Hum Reprod (2008) 23(4):885–8. doi: 10.1093/humrep/den008 18263638

[25] Liu Y Ye XY Chan C. The Association Between Endometrial Thickness and Pregnancy Outcome in Gonadotropin-Stimulated Intrauterine Insemination Cycles. Reprod Biol Endocrinol (2019) 17(1):14. doi: 10.1186/s12958-019-0455-1 30674305 PMC6345006

[26] Maher MA Abdelaziz A Shehata YA. Effect of Follicular Diameter at the Time of Ovulation Triggering on Pregnancy Outcomes During Intrauterine Insemination. Int J Gynaecol Obstet (2017) 139(2):174–9. doi: 10.1002/ijgo.12291 28771718

[27] Weiss NS van Vliet MN Limpens J Hompes PGA Lambalk CB Mochtar MH. Endometrial Thickness in Women Undergoing IUI With Ovarian Stimulation. How Thick Is Too Thin? A Systematic Review and Meta-Analysis. Hum Reprod (2017) 32(5):1009–18. doi: 10.1093/humrep/dex035 28333207

