# Supplementary figures and images for: The Effect of Spontaneous LH Surges on Pregnancy Outcomes in Patients Undergoing Letrozole-HMG IUI: A Retrospective Analysis of 6,285 Cycles
Source: Front Endocrinol (Lausanne). 2022 May 4;13:880538. doi: 10.3389/fendo.2022.880538 (PMC9114301; doi:10.3389/fendo.2022.880538)

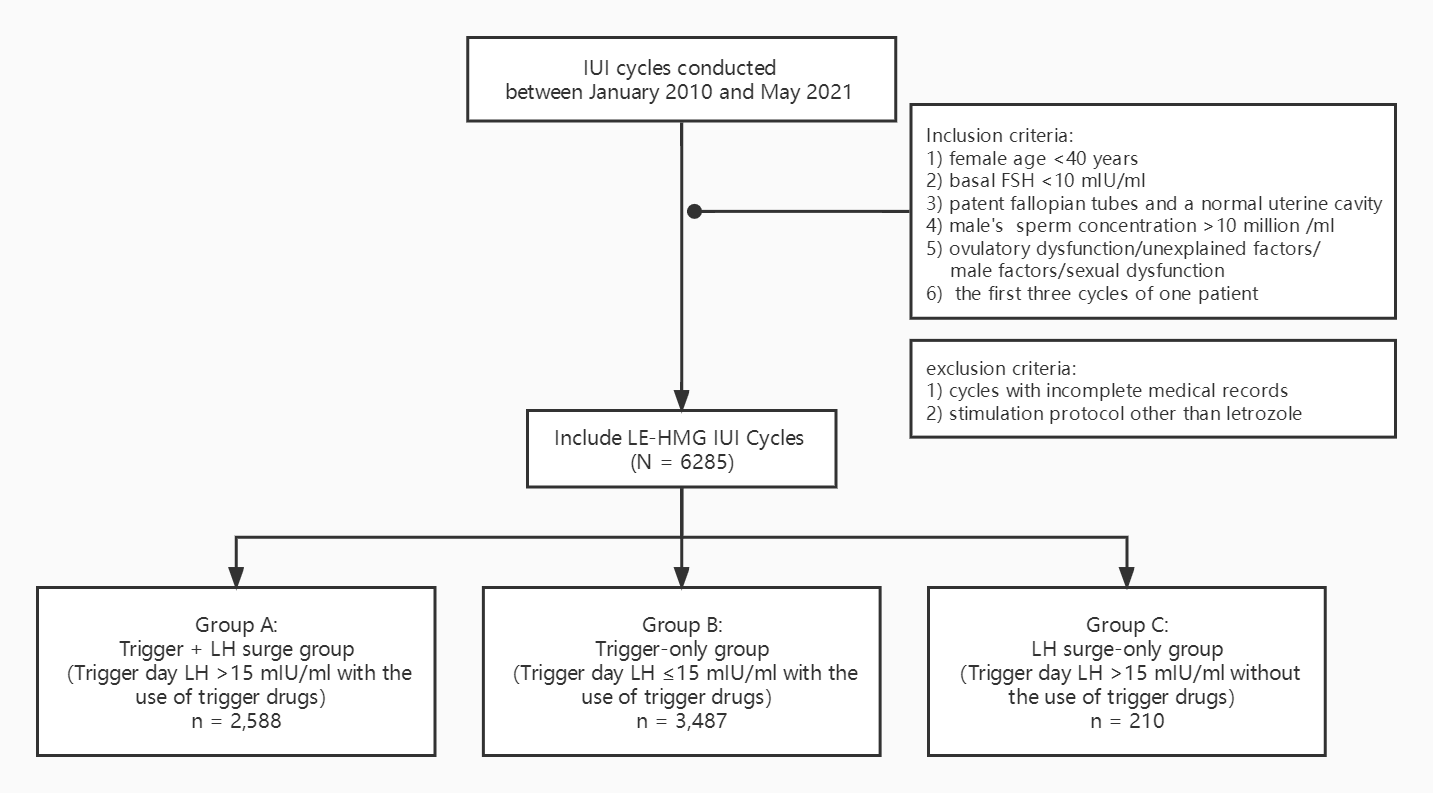

Supplement: Supplementary file 1 [file Image_1.jpeg]
